# Evaluation of the effectiveness of X-ray protective aprons in experimental and practical fields

**DOI:** 10.1007/s12194-013-0246-x

**Published:** 2013-12-13

**Authors:** Hiroshige Mori, Kichiro Koshida, Osamu Ishigamori, Kosuke Matsubara

**Affiliations:** 1 Department of Radiology, Hokkaido Social Insurance Hospital, 1-8-3-18 Nakanoshima, Toyohira, Sapporo, Hokkaido 062-8618 Japan; 2 Department of Quantum Medical Technology, Division of Health Sciences, Graduate School of Medical Science, Kanazawa University, 5-11-80 Kodatsuno, Kanazawa, Ishikawa 920-0942 Japan; 3 School of Health Sciences, College of Medical, Pharmaceutical and Health Sciences, Kanazawa University, 5-11-80 Kodatsuno, Kanazawa, Ishikawa 920-0942 Japan

**Keywords:** Analysis of covariance, Computed tomography, Interventional radiology, Protective apron, Radiation protection, X-ray transmission rates

## Abstract

Few practical evaluation studies have been conducted on X-ray protective aprons in workplaces. We examined the effects of exchanging the protective apron type with regard to exposure reduction in experimental and practical fields, and discuss the effectiveness of X-ray protective aprons. Experimental field evaluations were performed by the measurement of the X-ray transmission rates of protective aprons. Practical field evaluations were performed by the estimation of the differences in the transit doses before and after the apron exchange. A 0.50-mm lead-equivalent-thick non-lead apron had the lowest transmission rate among the 7 protective aprons, but weighed 10.9 kg and was too heavy. The 0.25 and 0.35-mm lead-equivalent-thick non-lead aprons differed little in the practical field of interventional radiology. The 0.35-mm lead apron had lower X-ray transmission rates and transit doses than the 0.25-mm lead-equivalent-thick non-lead apron, and each of these differences exceeded 8 % in the experimental field and approximately 0.15 mSv/month in the practical field of computed tomography (*p* < 0.01). Therefore, we concluded that the 0.25-mm lead-equivalent-thick aprons and 0.35-mm lead apron are effective for interventional radiology operators and computed tomography nurses, respectively.

## Introduction

Recently, attention has focused on orthopedic injuries attributed to the weight of X-ray protective aprons [1–3]. To resolve this problem, lighter aprons, made of composite materials, have been developed successfully [4–6]. These composite materials include several heavy metals such as copper, yttrium, tin, antimony, barium, tungsten, and lead [7–9]. However, manufacturers have not adequately released information about these composites [9–11].

The figures of merit of these protective aprons are commonly expressed as lead-equivalent thicknesses, which are measured only for specific X-ray energies [10, 11]. However, various X-ray energies are used in workplaces [9, 10]. There is also a difference in X-ray attenuation between pure lead and composite materials, which is determined by X-ray energies [9]. Therefore, the X-ray transmission rates of protective aprons, which are often measured at optional energies, differ among manufacturers, despite having the same lead-equivalent thicknesses [10, 11].

Differences in lead-equivalent thicknesses or X-ray transmission rates among protective aprons are not always reflected in the radiation fields of workplaces: practical fields. For example, in interventional cardiology, no significant difference in X-ray shielding performance was reported between 0.25- and 0.35-mm lead-equivalent-thick non-lead aprons [12]. However, there have been few practical evaluation studies in workplaces [13].

Here, we evaluated the effects of personal exposure reduction in experimental and practical fields upon exchanging the X-ray protective apron type worn by medical staff. The experimental field evaluation was performed by the measurement of the X-ray transmission rates of protective aprons. The practical field evaluation was performed by the estimation of the differences in the transit doses before and after the apron exchange, with the values measured by individual monitoring. Thus, we aim to discuss the effectiveness of X-ray protective aprons.

## Materials and methods

We researched the effects of exposure reduction before and after the exchange of the X-ray protective apron types as follows:

- Exchanging 0.25-mm lead-equivalent-thick non-lead aprons for 0.35-mm lead-equivalent-thick non-lead aprons, for the first and second abdominal interventional radiology (IVR) operators
- Exchanging 0.25-mm lead aprons for 0.50-mm lead-equivalent-thick non-lead aprons, for interventional cardiology operators
- Exchanging 0.25-mm lead-equivalent-thick non-lead aprons for 0.35-mm lead aprons, for nurses in a workplace where computed tomography (CT) is performed

Table 1 shows the specifications and use conditions of the X-ray protective aprons.

**Table 1:** Specifications and use conditions of the X-ray protective aprons. The upper and lower aprons for each case are the types of protective aprons used before and after the exchange

Model	Maker	Weight	Lead		Medical X-ray apparatus used in workplaces
			Lead or not^a^	Nominal thickness^b^	
Case a: Abdominal interventional radiology operators					
*First operator*					
ALG-L	Hoshina	2.7 kg	(−)	0.25 mm	Infinix Celeve VC
ALG-L	Hoshina	3.6 kg	(−)	0.35 mm	Toshiba Medical Systems
*Second operator*					
PGC-L	Hoshina	2.9 kg	(−)	0.25 mm	Infinix Celeve VC
PGC-L	Hoshina	3.8 kg	(−)	0.35 mm	Toshiba Medical Systems
Case b: Interventional cardiology operator					
DLC-25L	Maeda	3.6 kg	(+)	0.25 mm	INNOVA 2000
LP-EA68	AADCO Medical	10.9 kg	(−)	0.50 mm	GE Healthcare Japan
Case c: Computed tomography nurses					
PGC-L	Hoshina	2.9 kg	(−)	0.25 mm	LightSpeed VCT scanner with 62 rows of detector elements
HF2-35L	Maeda	5.4 kg	(+)	0.35 mm	GE Healthcare Japan

Hoshina, Maeda, and GE Healthcare Japan: Tokyo, Japan

AADCO Medical: Rondolph Vermont, USA. Toshiba Medical Systems: Tochigi, Japan

^a^‘Lead or not’ expresses whether an X-ray protective apron involves lead ‘(+)’, or not ‘(−)’

^b^‘Nominal Thickness’ expresses the nominal lead-equivalent thickness of an X-ray protective apron

We tested the statistical differences in X-ray transmission rates and transit doses before and after the apron exchange in the above cases. If there were statistical differences, we computed the estimated differences statistically. We compared the statistical results of these X-ray transmission rates and transit doses.

### Figures of merit of the X-ray protective aprons

We measured the lead-equivalent thicknesses of the X-ray protective aprons as figures of merit. Currently, there are various lead-equivalent thickness evaluation methods [10, 11, 14]. We adopted a computational method from an apron attenuation formula [14], because it is possible to re-inspect lead-equivalent thicknesses easily in all facilities with only aluminum filters, which are easier to acquire than lead filters.

First, with aluminum filters, we measured the half-value layer of the primary X-rays and computed their effective energy. Second, we computed the lead attenuation coefficient, *μ*_Pb_ from the effective energy of the primary X-rays [15], considering that the attenuation coefficient is a function of photon energy. Third, we measured the doses through and without protective aprons, I′ and I. Last, we calculated the apron’s lead-equivalent thickness, *d*_apron_ by substituting *μ*_Pb_, I′ and I for the following apron attenuation formula:

1 $${\text{I}}^{{\prime }} = {\text{I}} \times {\text{e}}^{{ - \mu_{\text{Pb}} \,\times\, d_{\text{apron}}}} ,$$

2 $$d_{\text{apron}} = - \frac{1}{{\mu_{\text{Pb}} }} \times \ln \frac{{{\text{I}}^{{\prime }} }}{\text{I}}.$$

**Fig. 1:**
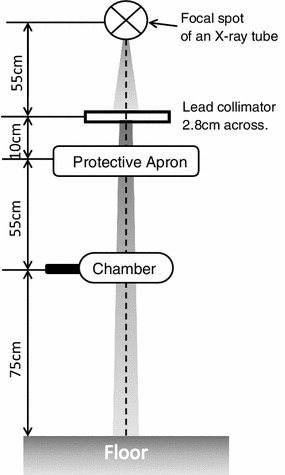
Geometry of an experimental field for measuring the lead-equivalent thicknesses and X-ray transmission rates of protective aprons

The medical X-ray apparatus used in this study was a DRX-3724HD X-ray tube with KXO-80G inverter-type high-potential generators (Toshiba Medical Systems, Tochigi, Japan), with an inherent filtration of 1.1-mm aluminum-equivalent thickness and an additional filtration of 2.7-mm aluminum-equivalent thickness. An ionization chamber, the DC300 3-cc thimble reference chamber (Wellhöfer, Schwarzenbruck, Germany), was interfaced with a RAMREC1500B dosimeter (Toyomedic, Tokyo, Japan). A 2.8-cm-diameter lead collimator was used for narrowing the X-ray beam. Figure 1 shows the geometries of these materials and the X-ray protective aprons. Aluminum filters with a fineness of 99.99 % for measuring the half-value layer were set at a 30-cm distance from the focal spot of the X-ray tube.

Primary X-rays were generated at 250 mA, 50 ms, and 120 kVp. In addition, for adjusting the effective energy of the primary X-rays to approximately 60 keV [14], an additional filter comprising 2.0-mm aluminum-equivalent and 0.2-mm copper-equivalent thicknesses was set at a 30-cm distance from the focal spot of the X-ray tube.

### Experimental field evaluation of X-ray protective aprons

#### Effective energy of primary X-rays used in an experimental field

We computed the effective energy of the primary X-rays used in an experimental field by measuring the half-value layer of the medical X-ray apparatus.

The half-value layer measurement of the primary X-rays was performed with the same materials as in Sect. 2.1, although the additional aluminum–copper filter was not used. The aluminum filter geometry for measurement of the half-value layer, a lead collimator to narrow the X-ray beam, and an ionization chamber were set at distances of 30, 55, and 180 cm, respectively, from the focal spot of the X-ray tube.

Primary X-rays were generated at 200 mA and 36 ms. We measured the half-value layers of the primary X-rays at 5 tube potentials: 50, 60, 80, 100, and 120 kVp. The effective energy of the primary X-rays was computed from the measured half-value layers [15].

#### X-ray transmission rates of protective aprons in an experimental field

The X-ray transmission rate of a protective apron, *T*, is an index that estimates the effect of exposure reduction in a practical field, and is given as follows:

3 $$T = \frac{{{\text{I}}^{{\prime }} }}{\text{I}} \times 100.$$

There are two measurement methods for the X-ray transmission rate with the narrow primary X-ray beam or the broad scatter X-ray beam [11, 16]. In this study, we adopted the narrow primary X-ray beam because the used ionization chamber volume was too small to use the broad scatter X-ray beam.

We measured the X-ray transmission rates of the X-ray protective aprons with the above formula (3) and the narrow beam. X-ray transmission rate measurements were performed with the materials and geometry (Fig. 1) of Sect. 2.1, although filters were not used. Primary X-rays used the same tube current and potentials as in Sect. 2.2.1, but the exposure time was 50 ms. We performed analysis of variance (ANOVA) to compare the X-ray transmission rates between the protective aprons in cases a, b, and c because of the two-way layout design with the five effective energies of primary X-rays per apron. Microsoft Office Excel 2007 Service Pack 3 software was used (Microsoft, Washington, USA). If there was a statistically significant difference by ANOVA, we estimated the difference before and after apron exchange.

### Practical field evaluation of X-ray protective aprons

#### Transit doses of X-ray protective aprons in a practical field

Medical staff occupational exposure was managed with personal dosimeter readings in a practical field. The effect of the exposure reduction in an X-ray protective apron ought to be reflected in the individual monitoring results. However, we could not merely compare exposure doses before and after apron exchange, because the working hours (i.e., the exposed doses to aprons) differed before and after apron exchange. Accordingly, we adopted an analysis of covariance (ANCOVA) to evaluate the effect of the X-ray protective apron exchange in a practical field.

Analysis of covariance is a general linear model-based statistical technique that has been presented as an extension of regression analysis and ANOVA [17]. ANCOVA is used for examining one-way layout design with the covariate as a nuisance factor. The covariate is the extraneous variable that influences each level’s quantitative variable at one factor. Using the quantitative variable as a dependent variable, the regression line is given as follows:

4 $$A_{i} :y_{ij} = \alpha_{i} + \beta_{i} x_{ij} \quad (j = 1, \, 2, \, 3, \ldots ,n),$$

where *A*_*i*_ is a level, called the qualitative independent variable, *y*_*ij*_ is the quantitative variable, called a dependent variable, *α*_*i*_ is a constant term, *β*_*i*_ is the inclination, and *x*_*ij*_ is the covariate, called a quantitative independent variable. *α*_*i*_ and *β*_*i*_ are not simply calculated at the general linear model regression analysis, but are calculated from the correlation of *x*_*ij*_ with *y*_*ij*_, called a covariance [17, 18]. ANCOVA is performed among quantitative variable levels with the residual error between the observed dependent variable and the predicted dependent variable from formula (4). Therefore, we can control the covariate-induced variance and increase the statistical precision to detect the differences among levels at one factor.

In ANCOVA, there are 2 prerequisite conditions for which nothing is the significant interaction between the qualitative and quantitative independent variables:

5 $$\beta_{1} = \beta_{2} = \beta_{3} = \cdots = \beta_{n} ,$$

and there is a significant linear relationship between a quantitative independent variable and a dependent variable:

6 $$\beta_{i} \ne 0 \quad \left( {i = 1, \, 2, \, 3, \ldots ,n} \right).$$

The statistical hypothesis (5) is not rejected by the *F* test and is called a regressive parallelism test. The statistical hypothesis against the alternative hypothesis (6) is rejected by the *F* test and is called a regressive significant test.

When ANCOVA was performed in this study, it was possible to remove the variance of the exposed doses to aprons as a nuisance factor from the variance of the transit doses through aprons, because exposed doses are covariates that influence transit doses. Accordingly, we can compare the differences in the transit doses among several apron types in a practical field without the influence of individual operation times before and after apron exchange.

From individual monitoring results with personal dosimeters, we estimated the difference in transit doses between the protective aprons in cases a, b and c. Individual monitoring was performed monthly with glass badges (Chiyoda Technol, Tokyo, Japan). Personal dosimeters were worn at the collar level above the protective apron and at the body level beneath the protective apron. The monthly measured collar level value, *H*_P_(10)_collar/month_, and the monthly measured body level value, *H*_P_(10)_body/month_, were shown as personal dose equivalents, defined in the International Commission on Radiation Units and Measurements (ICRU) Report 51 [19] at a tissue depth of 10 mm. The examination period included 2 years before and 2 years after the apron exchange. To estimate the difference in transit doses between the protective aprons, we performed ANCOVA as described above. *H*_P_(10)_body/month_, the transit dose through the protective apron, was a quantitative variable. *H*_P_(10)_collar/month_, the exposed dose to the protective apron, was a covariate. When the X-ray protective apron type is expressed by *A*_*i*_, formula (4) is updated as follows:

7 $$A_{i} :H_{\text{P}} (10)_{\text{body/month,ij}} = \alpha_{i} + \beta_{i} \times H_{\text{P}} (10)_{\text{collar/month,ij}} \quad \left( {j = 1, \, 2, \, 3, \ldots ,12} \right).$$

The significant difference of *H*_P_(10)_body/month_, after excluding covariates, is the difference in the transit doses before and after apron exchange, Δ*H*_P_(10)_body/month_:

8 $$\varDelta H_{\text{P}} ( 10)_{{{\text{body}}/{\text{month}}}} = \left| {\alpha_{2} - \alpha_{1} } \right|.$$

where *α*_1_ and *α*_2_ are constant terms before and after apron exchange, estimated by formula (7). Microsoft Office Excel 2007 Service Pack 3 software was used (Microsoft, Washington, USA).

In addition, if there were statistical differences in cases a, b, and c, we calculated the decreased annual effective dose using Δ*H*_P_(10)_body/month_. The monthly effective dose, *E*_eff/month_, for inhomogeneous exposure is given as follows [20]:

9 $$E_{\text{eff/month}} = 0.11 \times H_{\text{P}} (10)_{\text{collar/month}} + 0.89 \times H_{\text{P}} (10)_{\text{body/month}} .$$

Because *H*_P_(10)_collar/month_ does not vary with apron exchange, the reduction in the annual effective dose, *E*_eff/year_, was obtained from the following equation:

10 $$\varDelta E_{\text{eff/year}} = 12 \times 0.89 \times \varDelta H_{\text{P}} (10)_{\text{body/month}} .$$

#### Dose reduction rate of protective aprons in a practical field

We performed a *t* test of the dose reduction rates of X-ray protective aprons in a practical field to re-inspect the ANCOVA results. The dose reduction rate of an X-ray protective apron, *r*_*ik*_, is given as follows:

11 $$r_{ik} = \frac{{H_{\text{P}} (10)_{{\text{body/month,}}k} }}{{H_{\text{P}} (10)_{{\text{collar/month,}}k} }} \times 100\quad (k = 1, \, 2, \, 3, \ldots ,12).$$

We compared the ANCOVA and this *t* test result for cases a, b, and c.

## Results

### Figure of merit of the X-ray protective aprons

Table 2 shows the measured lead-equivalent thicknesses of the X-ray protective aprons. The lead-equivalent thicknesses of the 2 lead aprons were almost their nominal thicknesses. However, the lead-equivalent thicknesses of the 5 non-lead aprons were lower than expected. The effective energy used for these measurements was 62.5 keV.

**Table 2:** Nominal and measured lead-equivalent thicknesses of the X-ray protective aprons

Model	Lead-equivalent thickness of protective aprons	
	Nominal value	Measured value
Case a: Abdominal interventional radiology operators		
*First operator*		
ALG-L	0.25 mm	0.20 mm
ALG-L	0.35 mm	0.31 mm
*Second operator*		
PGC-L	0.25 mm	0.21 mm
PGC-L	0.35 mm	0.29 mm
Case b: Interventional cardiology operator		
DLC-25L	0.25 mm	0.25 mm^a^
LP-EA68	0.50 mm	0.52 mm^b^
Case c: Computed tomography nurses		
PGC-L	0.25 mm	0.21 mm
HF2-35L	0.35 mm	0.34 mm^a^

The upper and lower aprons for each case are the types of protective aprons used before and after the exchange

^a^X-ray protective apron involving lead

^b^Measured value with an additional shield

### Experimental field evaluation of X-ray protective aprons

**Fig. 2:**
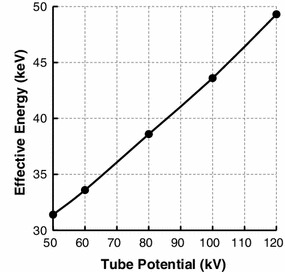
Relationship between the tube potential and the effective energy of the primary X-rays in an experimental field

Figure 2 shows the relationship between the tube potential and the effective energy of the primary X-rays in an experimental field. When the tube potential was varied from 50 to 120 kVp, the effective energy of primary X-rays was varied from 31.4 to 49.3 keV.

**Fig. 3:**
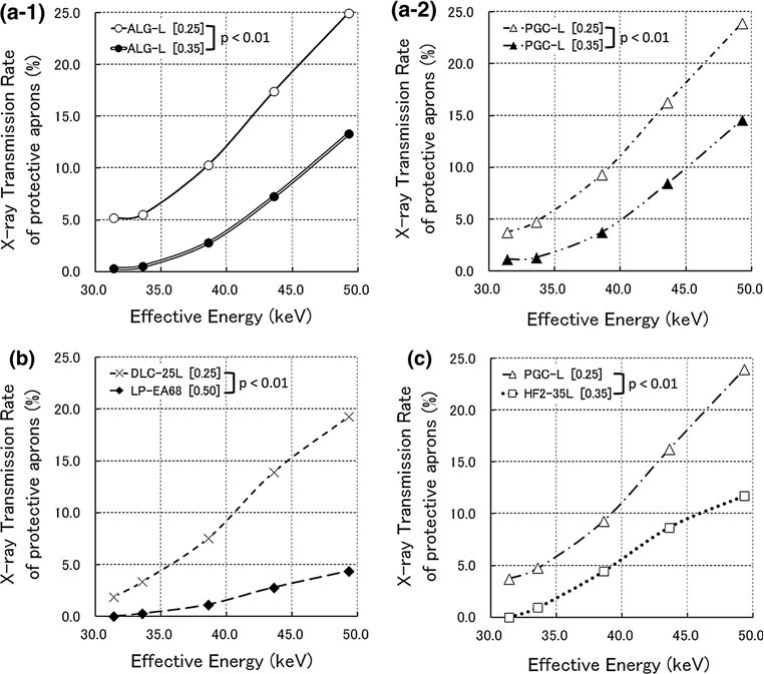
Relationship between effective energy and X-ray transmission rates of protective aprons in an experimental field. ‘[ ]’ in figures expresses the lead-equivalent thicknesses of X-ray protective aprons. **a-1** Comparison of protective apron types before and after exchange for the first abdominal interventional radiology operator. **a-2** Comparison of protective apron types before and after exchange for the second abdominal interventional radiology operator. **b** Comparison of protective apron types before and after exchange for the interventional cardiology operator. **c** Comparison of protective apron types before and after exchange for computed tomography nurses

Figure 3 shows the relationship between effective energy and X-ray transmission rates of protective aprons. The X-ray transmission rate increased along with the effective energy. There were significant differences in X-ray transmission rates after apron exchange in all Sect. 2 cases (*p* < 0.01). There was also a synergistic effect between effective energy and X-ray transmission rates of protective aprons in all Sect. 2 cases (*p* < 0.01). The 0.50-mm lead-equivalent-thick non-lead apron had the lowest transmission rate among the 7 protective aprons.

**Fig. 4:**
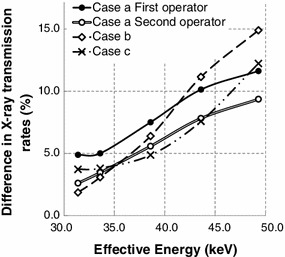
Difference in X-ray transmission rates before and after apron exchange in an experimental field. Cases a, b, and c upon exchange of the protective apron type are described at the beginning of Sect. 2

Figure 4 shows the estimated values of the difference in X-ray transmission rates before and after apron exchange in an experimental field. The difference in X-ray transmission rates before and after apron exchange increased with the effective energy.

### Practical field evaluation of X-ray protective aprons

In all Sect. 2 cases, the statistical hypothesis (5) was not rejected by the *F* test (*p* > 0.05), and the statistical hypothesis against the alternative hypothesis (6) was rejected by the *F* test (*p* < 0.01).

**Fig. 5:**
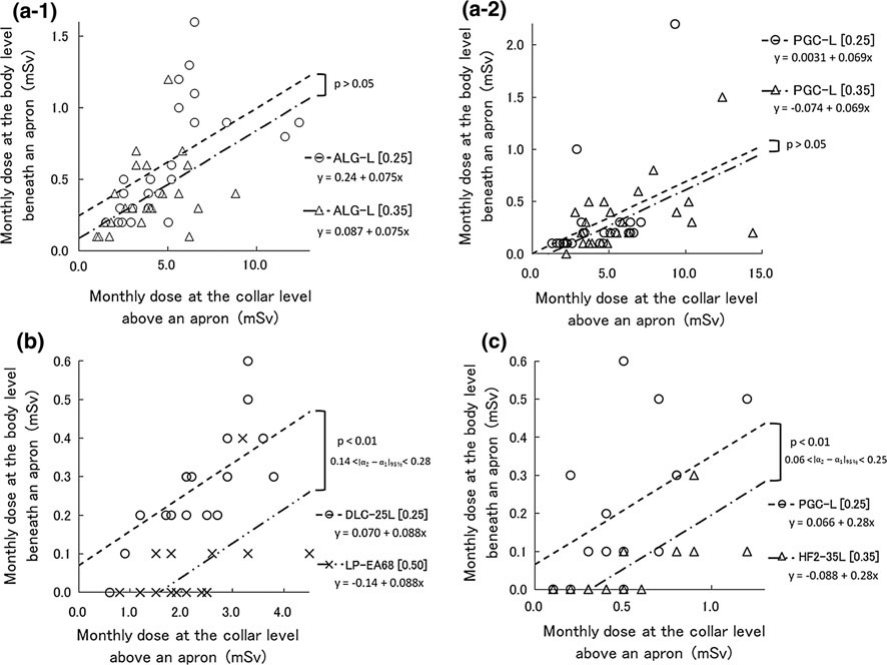
Relationship between the exposed doses to protective aprons ($H_{\text{P}} (10)_{\text{collar/month}}$) and the transmitted doses through protective aprons ($H_{\text{P}} (10)_{{{\text{body}}/{\text{month}}}}$) before and after the apron exchange in a practical field. These occupational doses express the personal dose equivalents, which are defined by International Commission on Radiation Units and Measurements (ICRU) Report 51 [19] in tissues at a depth of 10 mm. ‘[ ]’ and ‘$\left| {\alpha_{2} - \alpha_{1} } \right|_{95\% }$’ in figures express the lead-equivalent thicknesses of the X-ray protective aprons and the 95 % confidence interval, respectively. **a-1** Comparison between 0.25 and 0.35-mm lead-equivalent-thick non-lead aprons as worn by the first abdominal interventional radiology operator. **a-2** Comparison between 0.25 and 0.35-mm lead-equivalent-thick non-lead aprons as worn by the second abdominal interventional radiology operator. **b** Comparison between 0.25-mm lead apron and 0.50-mm lead-equivalent-thick non-lead apron as worn by the interventional cardiology operator. **c** Comparison between 0.25-mm lead-equivalent-thick non-lead apron and 0.35-mm lead apron as worn by computed tomography nurses

Figure 5 shows the relationship between the exposed doses to protective aprons and the transit doses through protective aprons before and after apron exchange. There were no significant differences between transit doses in case a of Sect. 2 (Fig. 5a). However, there were significant differences between transit doses in cases b and c of Sect. 2 In case b of Sect. 2 (Fig. 5c), the 0.50-mm lead-equivalent-thick non-lead apron had a lower transit dose of 0.21 mSv per month than the 0.25-mm lead apron (*p* < 0.01). In case c of Sect. 2 (Fig. 5d), the 0.35-mm lead apron had a lower transit dose of 0.15 mSv per month than the 0.25-mm lead-equivalent-thick non-lead apron (*p* < 0.01). The reductions in the annual effective dose were 2.2 mSv in case b of Sect. 2 and 1.6 mSv in case c of Sect. 2.

**Fig. 6:**
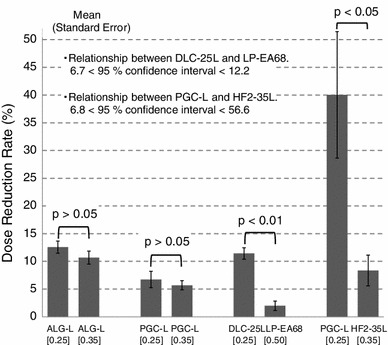
Difference in the dose reduction rate before and after the exchange of protective apron types in a practical field. ‘[ ]’ in a figure expresses the lead-equivalent thicknesses of the X-ray protective aprons

Figure 6 shows the *t* test results for the dose reduction rates for all cases in Sect. 2. The *t* test results agreed with the ANCOVA regarding all Sect. 2 cases.

## Discussion

There were differences between the nominal and measured lead-equivalent thicknesses of protective aprons. The measured lead-equivalent thicknesses of the non-lead aprons were smaller than their nominal thicknesses. This is not due to losses in the lead-equivalent thicknesses of protective aprons. Because non-lead aprons include low atomic number substances (compared with pure lead), it appears that the lead-equivalent thicknesses of non-lead aprons decrease with exposure to hard radiation quality caused by an additional filter, as in this study [10, 14]. Accordingly, we think that the X-ray protective aprons used in this study satisfied their nominal X-ray shielding performance.

In all Sect. 2 cases, there were statistical differences in the X-ray transmission rates before and after apron exchange. However, those evaluation did not consider the difference between the experimental and practical fields. The experimental field used in Sect. 2.2.2 supposed that primary X-rays would enter at the front of the protective aprons, but the practical field used in Sect. 2.3.1 supposed that scattered X-rays would enter in every direction. Consequently, two uncertainties arose regarding practical field applications: the incident angle and energy of the scattered X-rays which irradiate the protective apron.

X-ray transmission rate measurements reportedly have an uncertainty of 5 % between used primary and scattered X-rays [16]. In the practical field, scattered X-rays often enter protective aprons in the lateral and oblique directions [21]. Because IVR especially makes frequently the incident angulation of the primary X-rays which irradiate the patient, the uncertainty of X-ray transmission rates would exceed 5 % in IVR. The X-ray transmission rates depend on the X-ray energy (Fig. 3). Because scattered X-rays do not always enter filters at a front angle during measurements of effective energy, the large uncertainty surrounding the X-ray transmission rate arises from the measurement of the scattered X-ray effective energy in the practical field. This is why applications of X-ray transmission rates to practical fields appear awkward.

In case a of Sect. 2, the effective energy of the scattered X-rays would be, at most, 40 keV according to the Ref. [22] regarding the X-ray energies of used apparatus. Considering uncertainty beyond 5 % above, the practical difference in X-ray transmission rates is assumed to be a few percentage points (Fig. 4). Because the exposed doses to protective aprons did not exceed 7.0 mSv per month (Fig. 5), a few percentages of the X-ray transmission rate would be approximately 0.1 mSv for the transit dose, which is the glass badge detection limit dose. Therefore, there was no apparent significant difference in the transit doses between the non-lead aprons with 0.25-mm lead-equivalent thicknesses and those with 0.35-mm lead-equivalent thicknesses in case a of Sect. 2.

In case b of Sect. 2, the effective energy of the scattered X-rays is estimated as 35–50 keV according to the Ref. [23] regarding the X-ray energies of used apparatus. In this effective energy range, we detected a difference of X-ray transmission rates of 5–15 % (Fig. 4). After apron exchange, the 0.50-mm lead-equivalent apron had a marked ability to decrease the X-ray transmission rates, compared with the other aprons (Fig. 3). In an experimental field, these characteristics of X-ray transmission rates appear to cause significant differences in transit doses in a practical field. However, the 0.50-mm lead-equivalent-thick non-lead apron weighed 10.9 kg (Table 1). Orthopedic spinal, hip, knee, and ankle injuries have been observed with X-ray protective aprons of ≥5.6 kg [3]. Although the International Commission on Radiological Protection publications do not provide a reference description for case b of Sect. 2, the National Council on Radiation Protection and Measurements has advised that all new facilities and practices should be designed to limit 10-mSv fractions of the annual effective doses [24]. The reduction in the annual effective dose in case b of Sect. 2, 2.2 mSv, did not exceed this 10-mSv standard. Therefore, we think that this 2.2-mSv reduction is not sufficient to expose the operator to the risk of orthopedic injuries. We insist that the 0.25-mm lead-equivalent-thick non-lead aprons are sufficient to protect IVR operators. We recommend improving some protective devices rather than wearing 0.50-mm lead-equivalent non-lead aprons if additional protective measures are necessary.

In case c of Sect. 2, the effective energy of the scattered X-rays would exceed 45 keV according to the Ref. [25] regarding the X-ray energies of used apparatus. With this highly effective energy, we detected a difference in the X-ray transmission rates above 8 % in an experimental field (Fig. 4). There was also a significant difference in transit doses of Sect. 3.3. Moreover, after apron exchange, the reduction in the annual effective dose, 1.6 mSv, was approximately half of the annual effective dose before apron exchange. However, the 0.35-mm lead-equivalent-thick lead apron after exchange added 2.5 kg in weight (Table 1). We think that the risk of orthopedic injuries is small because nurses in CT rooms wear X-ray protective aprons only for a few minutes while acquiring CT data. We suggest that 0.35-mm lead-equivalent-thick lead aprons are effective for nurses in CT rooms.

Finally, although the practical evaluation regarding the transit doses of protective aprons involves the uncertainty about the incident angle and energy of the scattered X-rays, such evaluation is convenient because individual monitoring results are usable. Moreover, the ANCOVA was as statistically precise as the *t* test with respect to the dose reduction rate (Fig. 6). Therefore, we propose that practical field evaluations regarding the transit doses of protective aprons should be very useful for feedback after apron exchange.

## Conclusion

In this paper, we examined the effectiveness of X-ray protective aprons in 3 cases of abdominal IVR, interventional cardiology, and CT. The 0.25-mm lead-equivalent-thick aprons were sufficiently effective for operators in IVR because there was little difference between the 0.25-mm and 0.35-mm lead-equivalent-thick aprons. The 0.50-mm lead-equivalent-thick non-lead apron was too heavy. The 0.35-mm lead apron was effective for CT nurses because of the effectiveness against high energy X-rays such as those of CT.

The transmission rate of protective aprons in an experimental field changes by approximately 20 % even in the narrow range of effective energies of 33–50 keV. When X-ray protective aprons are exchanged in the future, we recommend selecting the protective apron type by considering the energy of scattered X-rays in workplaces. If X-ray protective aprons have already been exchanged, we recommend an additional inspection regarding their effectiveness in the practical field, because the result will not always agree with those of experimental field evaluations.
